# Commentary: Early Risk Detection of Burnout: Development of the Burnout Prevention Questionnaire for Coaches

**DOI:** 10.3389/fpsyg.2019.02721

**Published:** 2019-12-04

**Authors:** Erik Lundkvist, Henrik Gustafsson, Markus Gerber, Carolina Lundqvist, Andreas Ivarsson, Daniel J. Madigan

**Affiliations:** ^1^Performance and Training Unit, The Swedish School of Sport and Health Sciences, Stockholm, Sweden; ^2^Faculty of Arts and Social Sciences, Karlstad University, Karlstad, Sweden; ^3^Department of Coaching and Psychology, Norwegian School of Sport Sciences, Oslo, Norway; ^4^Sport Science Section, Department of Sport, Exercise and Health, University of Basel, Basel, Switzerland; ^5^Division of Psychology, Department of Behavioural Sciences and Learning, Linköping University, Linköping, Sweden; ^6^Centre of Research on Welfare, Health and Sport, Halmstad University, Halmstad, Sweden; ^7^School of Sport, York St John University, York, United Kingdom

**Keywords:** burnout, fatigue, exhaustion disorder, clinical burnout, burnout measurement

## Introduction

In a recent volume of this journal, Schaffran et al. (2019) introduced the Burnout Prevention Questionnaire for Coaches (BPQ-C). Although we recognize the worthwhile efforts of Schaffran et al., we believe that there are several issues associated with this instrument. This commentary aims to expand on why we think the BPQ-C should not currently be used by practitioners and researchers to screen for burnout.

## Lack of Theoretical Foundations

With regards to theory, we have four concerns. First, Schaffran et al.'s definition (and subsequent measurement) of burnout is inadequate. Although several definitions of burnout exist, most researchers agree that burnout is a multidimensional construct and models share—at a minimum—an exhaustion component (Maslach et al., 1996; Raedeke and Smith, 2001; Shirom, 2005). This was recently highlighted by the World Health Organization (2018). While not everyone agrees with one exact definition, no well-recognized definition includes general stress, social stress, and amotivation (Maslach et al., 1996; Raedeke and Smith, 2001; World Health Organization, 2018).

Second, Schaffran et al. (2019) seem to conflate antecedents, outcomes, and the construct of burnout itself. This point is exemplified by the fact that chronic job stress has historically been defined as an essential burnout antecedent and not a defining characteristic of burnout (Maslach et al., 1996; Shirom, 2005). Therefore, using stress as a symptom of burnout and not as an antecedent is conceptually incorrect. Similarly, the dimension labeled “pre-burnout” includes fatigue. This is confusing because of the evident conceptual overlap with exhaustion, which is known as a defining characteristic of burnout (Grossi et al., 2015).

Third, an underlying assumption for the BPQ-C seems to be that there exist sequential events in the burnout process. It is therefore peculiar that the authors relied exclusively on cross-sectional data when they created and tested their instrument (Leiter and Maslach, 1999, 2003; Hendrix et al., 2000). Especially since the complexity regarding temporality between burnout dimensions has been discussed before both in sport and more generally (Shirom and Melamed, 2006; Martinent et al., 2016).

Our final conceptual concern is the reliance on statistical considerations (from analysis of one sample), instead of also considering theoretical aspects, when deciding on the inclusion/exclusion of certain BPQ-C dimensions (Study 2). This exploratory approach has been criticized in the burnout literature previously and heightens risks for conceptual overlap with other constructs (see Shirom and Melamed, 2006), as well as misunderstanding of temporal relations with emotional exhaustion (Moore, 2000). The problem with this procedure becomes particularly apparent in relation to the resources dimension which defines sleep as a resource that may buffer potential burnout. However, with the same logic, poor sleep quality could be seen as a part of “pre-burnout” since poor sleep quality is a well-known antecedent of burnout symptoms (Söderström et al., 2012). Thus, although the authors briefly acknowledge the complexity of sleep in relation to burnout in their discussion, they do not provide any guidelines based on sound empirical work and theory to explain how sleep scores in the BPQ-C should be interpreted.

## Utility as a Screening Tool

The authors claim that the BPQ-C has the potential to be used as a screening tool in practical settings to detect early signs of burnout, and to guide prevention strategies before a coach develops more serious burnout symptoms. Although a commendable goal, one well-known problem is that there is often no clear distinction between normal functioning and psychological illness. Evaluations of symptoms should be related to the context in which they appear as well as to contemporary values and economic forces present in society and politics (Kinderman et al., 2017). Furthermore, Schaffran and colleagues do not provide evidence that the BPQ-C can be used to adequately screen for clinical burnout. To do so, analyses such as receiver operating characteristic analysis (ROC), which tests the ability of measures to discriminate between individuals with and without a certain clinical characteristic, is necessary (Kraemer, 1992). Comparisons between a clinical and a healthy sample were not a part of the validation of the BPQ-C. In fact, no descriptive statistics of burnout are reported. This makes it even harder to determine variation and representability of the samples that were used. In addition, even when a clinical sample is compared with a healthy sample, discriminating between healthy and ill individuals is challenging as they show substantial overlap (Lundgren-Nilsson et al., 2012). Therefore, using screening tools without clinical interviews creates substantial risks of both false positives and negatives, a matter that has been neglected in the sport psychology literature and is not considered by Schaffran et al. in their article.

## Questionnaire Development

Our last point of criticism concerns the development and validation of the BPQ-C. Several guides on how to develop a psychological instrument are available. Although they differ, there are some common grounds when it comes to item development, scale development, and scale evaluation (MacKenzie et al., 2011; Boateng et al., 2018). Schaffran et al., however, only communicate a few of these suggested steps. When such a step is communicated, like confirmatory factor analysis (CFA), the high degree of similarity in subscale content suggests a redundancy of these factors. This issue is compounded by the fact that parallel analysis suggested a one-factor model (see Sample 1). A stepwise evaluation would have easily eliminated this in the item developing phase. Lastly, in the three-factor CFA, several residuals are correlated (see Figure 1 in Schaffran et al., 2019). The reasons for this needs to be communicated since correlating residuals comes with several risks (with capitalization of chance being the most prominent one), making replication in another sample unlikely (Landis et al., 2009).

## Discussion

This commentary provides several arguments as to why, in our opinion, the BPQ-C fails to meet several theoretical, clinical, and psychometric standards. Substantial work is therefore needed before the BPQ-C can be considered a valid measure. Instead, we recommend the use of existing validated measures of burnout. One example of such a measure is the Shirom Melamed Burnout Questionnaire (SMBQ; Kushnir and Melamed, 1992), which is based on Hobfoll's Conservation of Resources (COR) theory (Hobfoll, 1989). The SMBQ has been validated in both clinical and healthy samples (Lundgren-Nilsson et al., 2012). Another alternative would be the Maslach Burnout Inventory (Maslach et al., 1996), which follows the theoretical underpinnings provided by Maslach and colleagues (Maslach et al., 1996). The MBI has shown reasonable psychometric properties in previous research, and for which at least some evidence exists that the instrument is able to discriminate between clinical and healthy individuals. However, the latter only applies to the exhaustion subscale (Kleijweg et al., 2013). Here, we note, however, that using self-report measures as the only source for clinical screening may not be successful because of the overlap between clinical and healthy individuals. We therefore recommend that, wherever possible, self-report screening should be combined with clinical interviews (Lundgren-Nilsson et al., 2012; Kleijweg et al., 2013) and that the interpretation of self-reported burnout measures with clinical cut-offs should be made with caution.

## Author Contributions

EL is the main author who initiated the commentary, wrote the first draft, and has been editing all other contributors' drafts and comments. HG was involved in the design of the paper and has been commenting and editing the paper throughout the whole process. MG has been commenting on the paper and has contributed uniquely to the lack of theoretical foundations part. CL has been commenting on the paper and has contributed uniquely to the clinical screening tool part. AI has been commenting on the paper and has contributed uniquely on the part called questionnaire development. DM was involved in the initial scoping of the paper, has done English editing, and contributed uniquely on the lack of theoretical foundations part.

### Conflict of Interest

The authors declare that the research was conducted in the absence of any commercial or financial relationships that could be construed as a potential conflict of interest.

## References

[B1] Boateng G. O. Neilands T. B. Frongillo E. A. Melgar-Quiñonez H. R. Young S. L. (2018). Best practices for developing and validating scales for health, social, and behavioral research: a primer. Front. Public Health 6:149. 10.3389/fpubh.2018.00149 29942800 PMC6004510

[B2] Grossi G. Perski A. Osika W. Savic I. (2015). Stress-related exhaustion disorder–clinical manifestation of burnout? A review of assessment methods, sleep impairments, cognitive disturbances, and neuro-biological and physiological changes in clinical burnout. Scand. J. Psychol. 56, 626–636. 10.1111/sjop.12251 26496458

[B3] Hendrix A. E. Acevedo E. O. Hebert E. (2000). An examination of stress and burnout in certified athletic trainers at Division IA universities. J Athletic Train. 35:139. PMC1323409 16558622

[B4] Hobfoll S. E. (1989). Conservation of resources: a new attempt at conceptualizing stress. Am. Psychol. 44:513. 2648906 10.1037//0003-066x.44.3.513

[B5] Kinderman P. Allsopp K. Cooke A. (2017). Responses to the publication of the American Psychiatric Association's DSM-5. J. Hum. Psychol. 57, 625–649. 10.1177/0022167817698262

[B6] Kleijweg J. H. Verbraak M. J. Van Dijk M. K. (2013). The clinical utility of the Maslach Burnout Inventory in a clinical population. Psychol. Assess. 25:435. 10.1037/a0031334 23356679

[B7] Kraemer H. C. (1992). Evaluating Medical Tests: Objective and Quantitative Guidelines, Vol. 26. Newbury Park, CA: Sage publications.

[B8] Kushnir T. Melamed S. (1992). The Gulf War and its impact on burnout and well-being of working civilians. Psychol. Med. 22, 987–995. 1488493 10.1017/s0033291700038551

[B9] Landis R. Edwards B. Cortina J. (2009). Correlated residuals among items in the estimation of measurement models, in Statistical and Methodological Myths and Urban Legends: Doctrine, Verity, and Fable in the Organizational and Social Sciences, eds Lance C. E. Vanderberg R. J. (Abingdon: Taylor & Francis), 195–214.

[B10] Leiter M. P. Maslach C. (1999). Six areas of worklife: a model of the organizational context of burnout. J. Health Hum. Serv. Admin. 21, 472–489. 10621016

[B11] Leiter M. P. Maslach C. (2003). Areas of worklife: a structured approach to organizational predictors of job burnout, in Emotional and Physiological Processes and Positive Intervention Strategies, eds Ganster D. C. Perrewé P. L. (Bingley: Emerald Group Publishing Limited), 91–134.

[B12] Lundgren-Nilsson Å. Jonsdottir I. H. Pallant J. Ahlborg G. (2012). Internal construct validity of the Shirom-Melamed Burnout questionnaire (SMBQ). BMC Public Health 12:1. 10.1186/1471-2458-12-1 22214479 PMC3307433

[B13] MacKenzie S. B. Podsakoff P. M. Podsakoff N. P. (2011). Constructmeasurement and validation procedures in MIS and behavioral research: integrating new and existing techniques. MIS Q. 35, 293–334. 10.2307/23044045

[B14] Martinent G. Louvet B. Decret J.-C. (2016). Longitudinal trajectories of athlete burnout among young table tennis players: a 3-wave study. J. Sport Health Sci. 10.1016/j.jshs.2016.09.003. [Epub ahead of print]. PMC7411113 32768130

[B15] Maslach C. Jackson S. E. Leiter M. P. (1996). Maslach Burnout Inventory Manual, Vol. 4. Palo Alto, CA: Consulting Psychologists Press.

[B16] Moore J. E. (2000). Why is this happening? A causal attribution approach to work exhaustion consequences. Acad. Manage. Rev. 25, 335–349. 10.5465/amr.2000.3312920

[B17] Raedeke T. D. Smith A. L. (2001). Development and preliminary validation of an athlete burnout measure. J. Sport Exerc. Psychol. 23, 281–306. 10.1123/jsep.23.4.281 28682196

[B18] Schaffran P. Kleinert J. Altfeld S. Zepp C. Kallus K. W. Kellmann M. (2019). Early risk detection of burnout: development of the burnout prevention questionnaire for coaches. Front. Psychol. 10:714. 10.3389/fpsyg.2019.00714 31024380 PMC6459965

[B19] Shirom A. (2005). Reflections on the study of burnout. Work Stress 19, 263–270. 10.1080/02678370500376649

[B20] Shirom A. Melamed S. (2006). A comparison of the construct validity of two burnout measures in two groups of professionals. Int. J. Stress Manage. 13:176 10.1037/1072-5245.13.2.176

[B21] Söderström M. Jeding K. Ekstedt M. Perski A. Åkerstedt T. (2012). Insufficient sleep predicts clinical burnout. J. Occup. Health Psychol. 17:175. 10.1037/a0027518 22449013

[B22] World Health Organization (2018). ICD-11 International Classification of Diseases for Mortality and Morbidity Statistics (11th Revision). Available online at: https://icd.who.int/browse11/l-m/en

